# Genetic Variants Associated with Breast Cancer Are Detected by Whole-Exome Sequencing in Vietnamese Patients

**DOI:** 10.3390/diagnostics15172187

**Published:** 2025-08-28

**Authors:** Nguyen Van Tung, Nguyen Thi Kim Lien, Le Duc Huan, Pham Cam Phuong, Bui Bich Mai, Nguyen Thi Hoa Mai, Tran Thi Thanh Huong, Phung Thi Huyen, Nguyen Van Chu, Tran Van Dung, Luu Hong Huy, Dong Chi Kien, Dang Van Manh, Duong Minh Long, Nguyen Ngoc Lan, Nguyen Thanh Hien, Ha Hong Hanh, Nguyen Huy Hoang

**Affiliations:** 1 Institute of Biology, Vietnam Academy of Science and Technology, Hanoi 100000, Vietnam; tungnv53@gmail.com (N.V.T.); ntkimlienibt@gmail.com (N.T.K.L.); leduchuankhtn@gmail.com (L.D.H.); hienmiu271095.vnu@gmail.com (N.T.H.); honghanhbio@gmail.com (H.H.H.); 2 Faculty of Biology, Graduate University of Science and Technology, Vietnam Academy of Science and Technology, Hanoi 100000, Vietnam; 3 The Nuclear Medicine and Oncology Center, Bach Mai Hospital, Hanoi 100000, Vietnam; phamcamphuong@gmail.com (P.C.P.); buibichmai.bvbm@gmail.com (B.B.M.); hoamaihmu@gmail.com (N.T.H.M.); 4 Viet Nam National Cancer Institute, K Hospital, Hanoi 100000, Vietnam; huongtran2008@gmail.com; 5 Department of Medical Oncology 6, K Hospital, Hanoi 100000, Vietnam; phungthihuyen@gmail.com (P.T.H.); dangmanh186@gmail.com (D.V.M.); 6 Quan Su Cytopathology Department, K Hospital, Hanoi 100000, Vietnam; chunv@bvk.org.vn (N.V.C.); longdm@bvk.org.vn (D.M.L.); 7 Department of International Collaboration and Research, K Hospital, Hanoi 100000, Vietnam; dungtranvk1011@gmail.com (T.V.D.); luuhonghuy95@gmail.com (L.H.H.); 8 Department of Medical Oncology 5, K Hospital, Hanoi 100000, Vietnam; jongkent@gmail.com; 9 Center for Gene and Protein Research, Hanoi Medical University, Hanoi 100000, Vietnam; ngoclana11108@yahoo.com

**Keywords:** Breast cancer, genetic variants, Vietnamese patients, whole-exome sequencing

## Abstract

**Background:** Breast cancer (BC) is the most common cancer and the leading cause of cancer death in women. Hereditary BC risk accounts for 25% of all cases. Pathological variants in known BC precursor genes explain only about 30% of hereditary BC cases, while the underlying genetic factors in most families remain unknown. Identifying hereditary cancer risk factors will help improve genetic counseling, cancer prevention, and cancer care. **Methods:** Here, we used whole-exome sequencing (WES) to identify genetic variants in 105 Vietnamese patients with BC and 50 healthy women. BC-associated variants were screened by the Franklin software and the criteria of the American College of Medical Genetics and Genomics (ACMG) and evaluated based on in silico analysis. **Results:** In total, 56 variants were identified in 37 genes associated with BC, including *ACVR1B*, *APC*, *AR*, *ARFGEF1*, *ATM*, *ATR*, *BARD1*, *BLM*, *BRCA1*, *BRCA2*, *CASP8*, *CASR*, *CHD8*, *CTNNB1*, *ESR1*, *FAN1*, *FGFR2*, *HMMR*, *KLLN*, *LZTR1*, *MCPH1*, *MLH1*, *MSH2*, *MSH3*, *MSH6*, *NF1*, *PMS2*, *PRKN*, *RAD54L*, *RB1CC1*, *RECQL*, *SLC22A18*, *SLX4*, *SPTBN1*, *TP53*, *WRN*, and *XRCC3* in 41 patients. Among them, 12 variants were novel, and 10 variants were assessed as pathogenic/likely pathogenic by ACMG and ClinVar. Variants of uncertain significance (VUS) were evaluated using in silico prediction software to predict whether they are likely to cause the disease in patients. **Conclusions:** This is the first WES study to identify BC-associated genetic variants in Vietnamese patients, providing a comprehensive database of BC susceptibility gene variants. We suggest using WES as a tool to identify genetic variants in BC patients for risk prediction and treatment guidance.

## 1. Introduction

According to Global Cancer Statistics 2022, breast cancer (BC) is the second most commonly diagnosed cancer, with approximately 2.3 million new cases and 670,000 deaths. BC is also the most common cancer and the leading cause of cancer death in women globally, accounting for 15.4% of all cancer deaths [1]. Although the highest incidence is in developed regions, many low and middle-income countries in Asia and Africa have the highest number of deaths (63% of the total) [2,3]. Some studies also show that BC appears earlier in Asian women (usually in their 40s–50s) than in Western women (in their 60s–70s) [4].

In addition, BC subtypes have been found to result in different treatment responses, drug resistance, and mortality rates in patients [5]. The Luminal A subtype typically exhibits features including aggressiveness, low proliferation, and a low-risk gene expression signature (GES). In contrast, the Luminal B subtype exhibits high aggressiveness, proliferation, and a high-risk GES; the HER2-enriched subtype typically exhibits intermediate to high proliferative features, and the TNBC subtype exhibits high aggressiveness and high proliferation [6]. The prognosis of the BC subtype also varies widely, with the Luminal A subtype having the best prognosis, followed by the Luminal B and HER2-enriched subtypes, and finally the TNBC subtype [7]. The TNBC subtype is thought to account for approximately 15–20% of BC cases, but has a shorter survival time and a mortality rate of up to 40% within the first 5 years due to its more aggressive nature [8]. The HER2-enriched subtype has a medium mortality, and lower mortality has been reported in the Luminal A and Luminal B subtypes [9].

The risk of disease is identified in 13–19% of patients with a first-degree relative with BC and increases when the relative is younger than 50 years [10,11]. Epigenetic and environmental factors are also considered potential triggers to increase the risk of disease [12]. A family history of ovarian cancer, especially those with pathogenic germline variants in the *BRCA1* and *BRCA2* genes, was identified in ∼20–25% of all hereditary BC cases [13]. Furthermore, pathogenic variants in other high- and moderate-risk genes, such as *TP53*, *CHEK2*, *ATM*, *STK11*, and *PALB2*, also lead to increased BC risk, indicating high complexity in BC predisposition [14]. Low-penetrance genes include *FGFR2*, *LSP1*, *MAP3K1*, *TGFB1*, *TOX3*, *RECQL*, *MUTYH*, *MSH6*, *NF1*, and *NBN* [7]. People with *BRCA1* and *BRCA2* mutations have a 10-fold increased risk of BC compared with women in the general population, and mutations in *CHEK2*, *ATM*, and *BRIP1* confer a two- to fourfold increased risk [15].

More than 35 genes have been reported; however, only a few of these genes have been established to have associations or demonstrated by both experimental and statistical methods [16,17]. Furthermore, despite efforts, variants in BC susceptibility genes are identified in <30% of BC cases with a family history or early onset [16,18]. This means that the underlying genetic factors of most BC cases remain unknown. Over the past few years, advances in next-generation sequencing (NGS) technology, particularly whole-exome sequencing (WES), have enabled the identification of pathogenic variants in many genetic diseases, including hereditary BC. Several novel BC susceptibility genes, such as *XRCC2*, *RINT1*, *RECQL*, and *FANCM*, have been identified by WES [18]. However, the small number of new proto-oncogenes revealed in these studies suggests that rare, or even exceptional, high- and moderate-penetrance variants may exist. Conversely, other forms of inheritance, such as recessive and oligogenic inheritance, need to be considered [19]. In this study, we performed WES on Vietnamese BC patients to expand the list of BC genes in different populations.

## 2. Materials and Methods

In total, 105 BC patients and 50 healthy women (with no family history of BC) were selected for WES. Control samples were selected based on the criteria of women aged >18 years, volunteering to participate in the study, and having no personal or family history of BC or other cancers. Patients ranged in age from 29 to 73 years, with a mean age of 49.3 ± 9.7 years. The clinical features and family history of cancer are described in Table 1. Patient identification information was encrypted and kept confidential in accordance with the provisions of the Declaration of Helsinki. This study was approved by the Ethics Committee of the Institute for Genomic Research (Approval Number 01-2021/NCHG-HDDD, 26 October 2021).

DNA was extracted from blood samples using a Qiagen DNA Blood Mini Kit (QIAGEN, Hilden, Germany) and used for WES on the Illumina sequencing system (Illumina, CA, USA). WES data with an average throughput depth of target regions of 150X, base quality thresholds with Q20 > 95% and Q30 > 90%, read alignment parameters, and variant caller filters with ReadPosRankSum < −8.0 were used for bioinformatic analysis. The BWA (version 0.7.17, http://bio-bwa.sourceforge.net/bwa.shtml, URL (accessed on 21 July 2025)), Picard (version 2.18.2, http://broadinstitute.github.io/picard/, accessed on 21 July 2025), GATK (version 3.4, https://www.broadinstitute.org/gatk/, accessed on 21 July 2025), and SnpEff (version 4.1, http://snpeff.sourceforge.net/SnpEff.html, accessed on 21 July 2025) software were used for subsequent analysis. The variants were first filtered using the Franklin software (https://franklin.genoox.com) and screened for pathogenicity variants based on the assessment criteria of the ACMG and ClinVar guidelines. Variants of uncertain significance (VUSs) were then further evaluated using in silico prediction software to predict the pathogenicity of the variants.

The pathogenicity of the VUSs were predicted using in silico prediction software for missense variants using the following tools: Bayes Del (https://bat.mpp.mpg.de/, accessed on 21 July 2025), DANN (https://cbcl.ics.uci.edu/public_data/DANN/, accessed on 21 July 2025), Fit Con (https://www.maudsleybrc.nihr.ac.uk/posts/2024/june/new-software-to-help-predict-individuals-genetic-risk-of-health-conditions/, accessed on 21 July 2025), Geno Canyon (https://zhaocenter.org/GenoCanyon_Index.html, accessed on 21 July 2025), Meta (http://asia.ensembl.org/info/genome/variation/prediction/protein_function.html, accessed on 21 July 2025; https://meta-analysis.com/pages/, accessed on 21 July 2025), Mutation Assesor (http://projects.sanderlab.org/), Mutation Taster (https://www.genecascade.org/MutationTaster2021/, accessed on 21 July 2025), Polyphen2 (http://genetics.bwh.harvard.edu/pph2/, accessed on 21 July 2025), Primate AI (https://newatlas.com/biology/primate-ai-breakthrough-predicting-human-diseases/, accessed on 21 July 2025), SIFT (https://sift.bii.a-star.edu.sg/, accessed on 21 July 2025), and Varity (https://genebe.net/hub/@genebe/varity/0.0.1, accessed on 21 July 2025). The splice variants were predicted by the Human Splicing Finder (HSF) (https://genomnis.com/) and MaxEnt Scan (http://hollywood.mit.edu/burgelab/maxent/Xmaxentscan_scoreseq.html, accessed on 21 July 2025).

## 3. Results

In this study, patients were divided into four groups: the Luminal A group (with ER+/Her2-, including 26 patients), the Luminal B group (with ER+/Her2+, including 32 patients), the HER2-enriched group (with ER-/Her2+, including 29 patients), and the TNBC group (with ER-/PR-/Her2-, including 18 patients) (Table 1) [20]. Patients in the Luminal A, Luminal B, and HER2-enriched groups had similar age ranges of 31 to 67 years (mean 50.1 ± 8.9), 37 to 73 years (mean 51.8 ± 9.1), and 33 to 68 years (mean 50.9 ± 8.9), respectively. Meanwhile, the TNBC group had a younger age of onset from 29 to 60 years (mean 43.9 ± 8.1).

Family history of first- and second-degree relatives with cancer was also investigated. The results showed that all patient groups had a family history of relatives with BC. The proportion of patients with relatives who had BC was highest in the TNBC group (22.2%), followed by the Luminal A (19.3%) and Luminal B (12.5%) groups, with the lowest in the HER2-enriched group (6.7%). The proportion of patients with relatives who had ovarian cancer was highest in the TNBC group (11.1%), with the rates in the Luminal A and B groups being 3.8% and 3.1%, respectively. The HER2-enriched group had no patients with relatives who had ovarian cancer. All patient groups had a family history of cervical cancer; the rates were not high, with the highest being 6.7% in the HER2-enriched group, followed by the TNBC group (5.6%) and the Luminal A group (3.8%), with the lowest in the Luminal B group (3.1%). Family history of lung cancer was also high in the Luminal A and B groups at 11.6% and 12.5%, respectively. In addition, patients with a family history of colorectal cancer and other cancers were also recorded. However, patients without a family history of cancer accounted for a high proportion in the groups, with the highest in the HER2-enriched group (69.4%), followed by the TNBC group (61.1%), and the lowest in the Luminal A and B groups at 53.9% and 56.3%, respectively.

**Figure 1:**
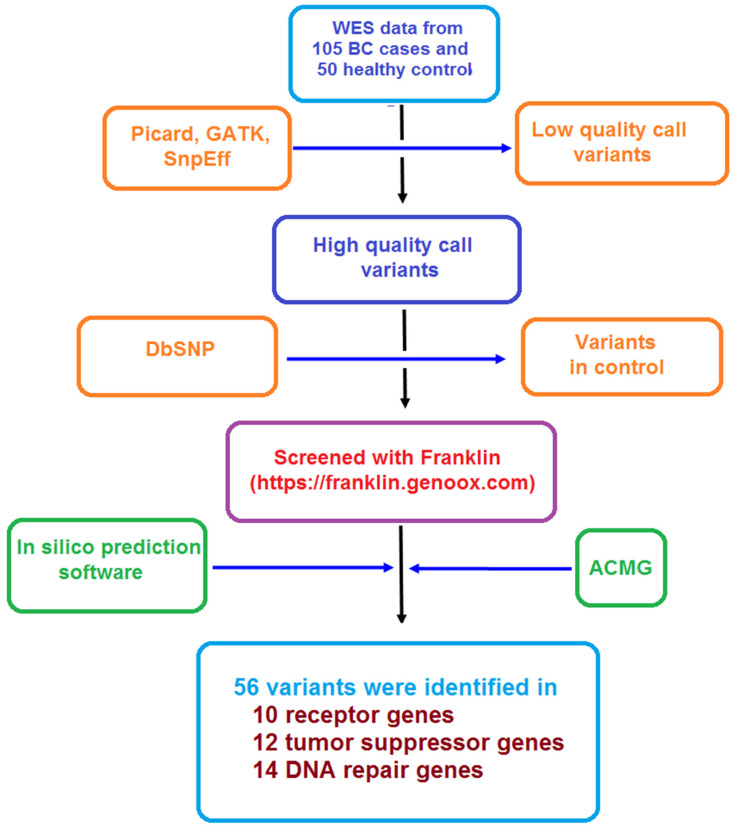
Bioinformatics screening strategy workflow for the candidate genes from the WES data.

We used WES to identify pathogenic variants in patients with BC; 56 variants in 37 genes were identified in 41 patients, including *ACVR1B*, *APC*, *AR*, *ARFGEF1*, *ATM*, *ATR*, *BARD1*, *BLM*, *BRCA1*, *BRCA2*, *CASP8*, *CASR*, *CHD8*, *CTNNB1*, *ESR1*, *FAN1*, *FGFR2*, *HMMR*, *KLLN*, *LZTR1*, *MCPH1*, *MLH1*, *MSH2*, *MSH3*, *MSH6*, *NF1*, *PMS2*, *PRKN*, *RAD54L*, *RB1CC1*, *RECQL*, *SLC22A18*, *SLX4*, *SPTBN1*, *TP53*, *WRN*, and *XRCC3* (Table 2, Figure 1). Among these, there were 10 receptor genes (*ACVR1B*, *AR*, *ARFGEF1*, *CASP8*, *CASR*, *CTNNB1*, *ESR1*, *FAN1*, *FGFR2*, and *PRKN)*, 12 tumor suppressor genes (*APC*, *BARD1*, *HMMR*, *KLLN*, *LZTR1*, *MCPH1*, *MLH1*, *NF1*, *RB1CC1*, *SLC22A18*, *SPTBN1*, and *TP53)*, and 14 DNA repair genes (*ATM*, *ATR*, *BLM*, *BRCA1*, *BRCA2*, *CHD8*, *MSH2*, *MSH3*, *MSH6*, *PMS2*, *RAD54L*, *RECQL*, *SLX4*, *WRN*, and *XRCC3).*

**Figure 2:**
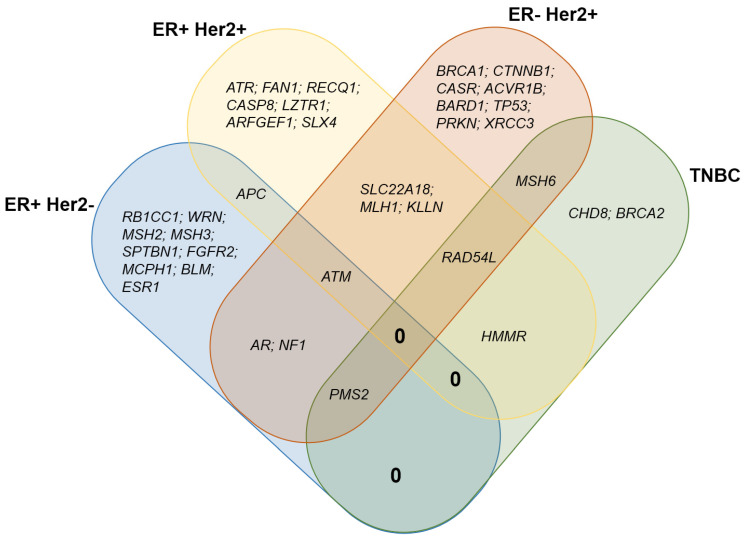
A Venn diagram showing the overlap and unique genes of each subtype. The ATM, PMS2, and RAD54L genes overlap between the following subtypes: Luminal A (ER+Her2-), Luminal B (ER+Her2+), and TNBC and Luminal B (ER+Her2+), HER2-enriched, and TNBC, respectively. The APC, AR, HMMR, KLLN, MLH1, MSH6, NF1, PMS2, and SLC22A18 genes overlap between two subtypes.

Five variants in *ARFGEF1*, *PMS2*, and *PRKN* were assessed as pathogenic by the ACMG, and five variants identified in *AR*, *CASR*, *FAN1*, *RECQL*, and *SLX4* were assessed as likely pathogenic (LP) by the ACMG. The proportions of patients with variants identified in the Luminal A, Luminal B, HER2-enriched, and TNBC groups were 34.6%, 24.1%, 44.8%, and 22.2%, respectively. A Venn diagram showing the overlap and unique genes of each subtype (Figure 2) has been built to show the ATM gene overlap between Luminal A (ER+Her2-), Luminal B (ER+Her2+), and TNBC subtypes; the PMS2 gene overlap between the Luminal A, HER2-enriched, and TNBC subtypes; and the RAD54L gene overlap between the Luminal B, HER2-enriched, and TNBC subtypes; the APC gene overlaps between Luminal A and Luminal B; the AR and NF1 gene overlaps between Luminal A and HER2-enriched; the KLLN, MLH1, and SLC22A18 gene overlaps between Luminal B and HER2-enriched; the HMMR gene overlaps between Luminal B and TNBC; and the MSH6 gene overlaps between HER2-enriched and TNBC subtypes.

Two patients carried three variants in different genes, and eleven patients carried two variants in different genes. Three patients carried variants in the *BRCA1* and *BRCA2* genes, of which one patient carried a pathogenic variant in the *BRCA2* gene, one patient carried a pathogenic variant in the *BRCA1* gene, and one patient carried both a VUS in the *BRCA1* gene and a pathogenic variant in another gene (Table 2). VUSs were evaluated by the predictive software, and 16 variants were assessed as pathogenic; 9 variants did not receive a consistent assessment of pathogenicity (Table 3). Of these, four patients, UK3, UK5, UB52, and UB62, carried two variants identified as pathogenic in the *SLX4* and *ATM* genes; the *RECQL* and *LZTR1* genes; the *ATM* and *MLH1* genes; and the *FGR2* and *PMS2* genes, respectively. Two patients, UB7 and UB35, had one pathogenic variant and one conflicting variant, identified in *BARD1* and *MSH6* and *PMS2* and *MSH6*, respectively. However, patients UB12, UB16, UB25, UB26, UB29, and UB44 carried variants in the genes *ATR* (c.4352G>A, p.Arg1451Gln), *KLLN* (c.250G>C, p.Gly84Arg), *SLC22A18* (c.604A>G, p.Ile202Val), *APC* (c.5290C>G, p.Gln1764Glu), *ATM* (c.3190A>G, p.Met1064Val), and *SLC22A18* (c.28A>C, p.Asn10His), which were predicted as benign. The results of the prediction of the splicing variants using the human splice finder (HSF) and MaxEnt Scan (Table 4) showed that variants in the *SPTBN1* (c.109+1G>T), *BLM* (c.3558+3A>G), and *MSH3* (c.3302+4A>C) genes resulted in the creation of a novel donor site and may be pathogenic in patients. However, the pathogenicity of variants in the *NF1* (c.888+5G>A), *HMMR* (c.146-4G>A), and *ATM* (c.2838+9C>T) genes was not determined in patients UB23, UB30, or UB48.

## 4. Discussion

BC is the most common type in women, with an incidence rate of ~31% (ranked first) and a mortality rate of 15% (ranked second) [21]. Although the cause and mechanisms of BC have not been fully determined, genetics is considered one of the most important factors, with familial cases and hereditary cases accounting for 15–20% and 5–10% of all cases, respectively [16,22,23]. People with a history of first-degree relatives have a significantly higher risk of developing BC than people with no family history. The risk is 4.3% for people with no family history and 8.1% for people with a family history of BC. If a first-degree relative is diagnosed with BC or bilateral BC before age 40, the risk is three to nine times higher than for people with no family history [24].

In our study, the TNBC subtype had the highest proportion of patients with a family history of BC and ovarian cancer (22.2% and 11.1%, respectively), followed by patients in the Luminal A and Luminal B subtypes, with rates of 19.3%/3.8%, and 12.5%/3.1%, respectively, with the lowest in the HER2-enriched subtype with 6.7% and 0% (Table 1). This result is similar to previous studies because the TNBC patients in our study had an average age of disease onset of 43.9 ± 8.1 years, which is lower than that of other subtypes. We used WES to identify pathogenic variants in patients with BC; 56 variants in 37 genes were identified in 41/105 patients (accounting for 39.1%). However, the variant detection rate was highest in the HER2-enriched subtype (44.8% with 13/29 patients), followed by the Luminal A subtype (34.6% with 9/26 patients), with the lowest in the Luminal B and TNBC subtypes, with rates of 24.1% (with 7/32 patients) and 22.2% (with 4/18 patients), respectively. We also identified eight patients (UK3, UK5, UB7, UB35, UB43, UB52, UB59, and UB62) carrying two variants in different genes. A polygenic model for cancer predisposition in these patients has been proposed and reviewed by many authors [16], with alleles of intermediate and low penetrance acting synergistically and playing a dominant role. In addition, the large number of relatives affected by different tumor types on both the maternal and paternal sides of these families may be a confounding factor in understanding the co-segregation phenotypes and outcomes. These genes have been reported to be associated with increased BC risk, therapy resistance, and prognosis in patients.

### 4.1. Receptor Genes

Receptor genes such as *ACVR1B (ALK4)*, *AR*, *ARFGEF1 (BIG1)*, *CASP8*, *CASR*, *CTNNB1*, *ESR1*, *FAN1*, *FGFR2*, and *PRKN* are associated with BC through their roles in cell signaling pathways, which play a role in many cellular processes (including cell growth, differentiation, and apoptosis) and epigenetic regulation [25]. Receptor genes play a role in BC, both as potential biomarkers and as therapeutic targets. The *AR* gene is found in the majority of BC, regardless of estrogen receptor (ER) status, and its expression may vary in different BC subtypes [26]. The *CASP8* gene is the first low-penetrance gene identified to be associated with BC risk and is a diagnostic and prognostic marker in BC [27,28,29]. The *CASR* gene is involved in BC development and progression, promoting proliferation and metastasis, although its role is complex and may vary depending on the context [30,31]. The *CTNNB1* gene and abnormal beta-catenin signaling are associated with BC development and progression. Ozcan et al. [32] showed an up-regulation associated with drug resistance in the ER+/Her2- patients. The results suggest that *CTNNB1* can be used as a powerful and effective predictor to guide chemotherapy decisions in ER+/Her2- patients at high risk of recurrence. Altiparmak-Ulbegi et al. [33] found that *CTNNB1* variants could be a potential biomarker for determining PTX resistance in the ER+/Her2- patients.

Variants in the *ESR1* gene can lead to endocrine therapy resistance and poorer survival, thus having predictive significance in influencing treatment efficacy and tumor progression [34,35,36,37,38]. Although variants in the *FAN1* gene have been identified in 14 BC and ovarian cancer patients in families with early-onset cancers, the association of these variants with increased BC risk has not been consistent [39]. Variants in the *FGFR2* gene result in tumor cell proliferation and survival but can also inhibit tumor growth and enhance p53-induced DNA damage signaling [40]. The *PRKN* gene is involved in BC tumor development and growth, and loss of Parkin expression due to promoter methylation may be used as a prognostic marker for BC survival [41].

### 4.2. Tumor-Suppressor Genes

Tumor-suppressor genes, including *APC*, *BARD1*, *HMMR (RHAMM)*, *KLLN*, *LZTR1*, *MCPH1 (BRIT1)*, *MLH1*, *NF1*, *RB1CC1 (FIP200)*, *SLC22A18*, and *SPTBN1*, have been found to increase the risk of disease and tumor development [42,43]. The *BARD1* gene is associated with a 17–30% higher risk of BC in *BARD1* variant carriers compared to the general population, especially the TNBC subtype. This gene plays a complex role in the development of BC, acting as a tumor suppressor and also exhibiting oncogenic properties [44]. The *HMMR* gene is associated with an increased risk of early-onset BC and contributes to cancer progression through the control of cell growth and cancer spread to other parts of the body [45]. *HMMR* plays a pivotal role as an oncogenic regulator in maintaining cell pluripotency and resistance to anticancer drugs [46], highlighting it as a potential target for therapeutic intervention. The *KLLN* gene regulates cell growth; overexpression leads to cell death, while inhibition leads to cell proliferation and BC progression [47].

Variants in *LZTR1* can disrupt the RAS/MAPK signaling pathways and lead to uncontrolled cell proliferation and tumor development [48]. Germline *NF1* variants leading to RAS activation and MAPK pathway activation increase the risk of BC, especially in women under 50 years of age, which may lead to an increased risk of cancer mortality [49]. *NF1* somatic variants are rare in primary cancers; are associated with poor prognosis and increased risk of recurrence [50]; have a high incidence of contralateral BC, poor survival [51], and BC progression; and contribute to endocrine therapy resistance [52,53]. Lower expression levels of *SLC22A18* have been reported to be associated with progression, recurrence, and poorer survival outcomes in BC patients [54]. The *SPTBN1* gene inhibits processes such as epithelial–mesenchymal transition (EMT), proliferation, and metastasis of cancer cells, which are associated with lower survival rates and unfavorable prognosis in BC patients [12].

### 4.3. DNA Repair Genes

DNA repair genes, including *ATM*, *ATR*, *BLM*, *CHD8*, *MSH2*, *MSH3*, *MSH6*, *PMS2*, *RAD54L*, *RECQL*, *SLX4 (FANCP)*, *WRN*, and *XRCC3*, which play important roles in homologous recombination DNA repair, maintenance of genomic integrity, and cell cycle regulation, are considered potential BC genes [55,56,57,58]. Variants in the *ATM* gene may lead to a slightly increased risk of BC [59]. The *BLM* gene is being investigated as a BC susceptibility gene and has been associated with survival following immunotherapy across multiple cancers [60,61]. The *CHD8* gene is associated with the development and progression of BC, especially in patients of the TNBC subtype [62], and is endowed with more nefarious pro-oncogenic capabilities [62,63]. *MSH6* and *PMS2* variants have been identified as associated with increased BC risk in individuals with a personal and/or family history of BC [64]. While the *RECQL* gene has been identified as the strongest susceptibility gene for BC [65], the *RAD54L* gene variant is known to be associated with the TNBC subtype [66]. Additionally, *RECQL* variants of unknown cause occur more frequently in patients with the Her2+ subtype than in patients with other subtypes [67].

Some limitations of our study arise from the WES method, which may have resulted in the omission of variants located in non-coding regions, copy number variants, and large genomic rearrangements associated with BC. In addition, the interpretation of variant effects is limited, especially in cases where patients carry multiple variants. However, our results provide data on genomic variants associated with BC in Vietnamese patients that provide a basis for prognosis and genetic counseling for affected families and suggest that WES is a useful tool for variant identification in the detection of disease mechanisms. A database of BC-associated variants is being built for use in software development to predict breast cancer risk in Vietnamese patients.

## 5. Conclusions

In summary, we are the first to perform WES analysis and identified 56 variants in 37 genes of Vietnamese patients with BC. The evaluation revealed that 43 variants were pathogenic in patients, providing a database of variants for understanding the pathogenesis and developing future treatment strategies. These findings demonstrate the importance of genetic testing, especially for those with a family history of BC, toward the development of more effective personalized medicine.

## Figures and Tables

**Table 1:** Clinical characteristics of patients in the study.

**Patient Characteristic**	**Clinical Breast Cancer Pathology Subtypes**			
	**Luminal A** **ER** **+** **/Her2-**	**Luminal B** **ER** **+** **/Her2** **+**	**HER2-Enriched** **ER-/Her2** **+**	**TNBC**
Patient No. (*n* = 105)	Average age is 49.3 ± 9.7 years old (range of age: 29–73)			
Mean age (SD), y (n)	50.1 ± 8.9 (n = 26)	51.8 ± 9.1 (n = 32)	50.9 ± 8.9 (n = 29)	43.9 ± 8.1 (n = 18)
Range of age at diagnosis of breast cancer (y)	31–67	37–73	33–68	29–60
Age of patient	Number of patients with/without variant			
<30				1 (0/1)
31–40	4 (1/3)	5 (4/1)	6 (3/3)	6 (3/3)
41–50	10 (6/4)	8 (0/8)	9 (4/5)	8 (1/7)
51–60	9 (2/7)	12 (3/9)	10 (5/5)	3 (0/3)
>60	3 (0/3)	7 (0/7)	4 (1/3)	
Total number of patients with variants (%)	9 (34.6)	7 (24.1)	13 (44.8)	4 (22.2)
Ovarian cancer				1
Thyroid cancer		1		
Skin cancer	1			
Family history, no. (%)				
Breast cancer	5 (19.3)	4 (12.5)	2 (6.7)	4 (22.2)
Ovarian cancer	1 (3.8)	1 (3.1)		2 (11.1)
Lung cancer	3 (11.6)	4 (12.5)		
Colorectal cancer	1 (3.8)	1 (3.1)	1 (3.4)	
Endometrial cancer	1 (3.8)	1 (3.1)	2 (6.7)	1 (5.6)
Other cancer	1 (3.8)	3 (9.4)	4 (13.8)	
No cancer family history	14 (53.9)	18 (56.3)	20 (69.4)	11 (61.1)

ER: estrogen receptor; TNBC: triple-negative breast cancer (ER-/PR-/Her2-).

**Table 2:** Variants identified in patients in this study.

Patient	Age of Onset/ Subtype	Gene	cDNA/Protein	dbSNP/ClinVar/ACMG	Family History
UK1	45 TNBC	*CHD8*	c.6472C>T p.Arg2158Cys	rs371915075/ VUS (PM2, PP2)	-
UK2	43 ER-/Her2+	*KLLN*	c.438delC p.Pro146fs	rs1405023663/ VUS (PM2)	-
UK3	37 ER+/Her2+	*SLX4*	c.4204C>T p.Gln1402*	Novel/ LP (PVS1)	breast
		*ATM*	c.5888A>G p.Asp1963Gly	rs1555110484/ VUS (PM2, PP3)	
UK5	60 ER+/Her2+	*RECQL*	c.820delT p.Cys274fs	Novel/ LP (PVS1, PM2)	-
		*LZTR1*	c.1646A>G p.Asp549Gly	rs762315626/ VUS (PM2, PP3)	
UK6	33 ER-/Her2+	*NF1*	c.4451C>G p.Ser1484Cys	Novel/ VUS (PM2, PP2)	-
UK12	53 ER+/Her2-	*WRN*	c.2977C>T p.Arg993Cys	rs749330842/ VUS (PM2)	-
UK16	47 ER+/Her2-	*MSH2*	c.2197G>A p.Ala733Thr	rs772662439/ VUS (PM2, PP3)	breast
UB3	39 TNBC	*RAD54L*	c.788G>A p.Gly263Glu	rs186059216/ VUS (PM2, PP3)	uterus
UB4	40 ER+/Her2+	*FAN1*	c.1822dupG p.Ala608fs	Novel/ LP (PVS1, PM2)	breast
UB6	54 ER-/Her2+	*CTNNB1*	c.1345C>T p.Arg449Cys	rs771596917/ VUS (PM2, PP2)	-
UB7	50 ER-/Her2+	*BARD1*	c.1620A>T p.Lys540Asn	rs747076015/ VUS (PM2)	-
		*MSH6*	c.944C>G p.Ser315Cys	rs63750491/ VUS (PM2)	
UB12	56 ER+/Her2+	*ATR*	c.4352G>A p.Arg1451Gln	rs371919176/ VUS (PM2, PP2)	other
UB16	73 ER+/Her2+	*KLLN*	c.250G>C p.Gly84Arg	rs3758479/ VUS (PM2)	other
UB17	54 ER+/Her2+	*MLH1*	c.776T>C p.Leu259Ser	rs56250509/ Pathogenic VUS (PM2, PP3)	other
UB18	64 ER-/Her2+	*TP53*	c.1024C>G p.Arg342Gly	Novel/ VUS (PM2)	-
UB19	51 ER+/Her2+	*RAD54L*	c.866G>A p.Ser289Asn	rs371268995/ VUS (PM2)	-
UB21	44 ER+/Her2-	*SPTBN1*	c.109+1G>T	Novel/ VUS (PM2)	ovary
		*NF1*	c.888+5G>A	rs556444929/ VUS (PM2)	
UB23	55 ER+/Her2-	*NF1*	c.888+5G>A	rs556444929/ VUS (PM2)	-
**Patient**	**Age of Onset/** **Subtype**	**Gene**	**cDNA/Protein**	**dbSNP/ACMG**	**Family History**
UB25	53 TNBC	*SLC22A18*	c.604A>G p.Ile202Val	rs758404808/ VUS (PM2)	breast
UB26	62 ER+/Her2+	*APC*	c.5290C>G p.Gln1764Glu	rs529543591/ VUS (PM2)	-
UB29	49 ER-/Her2+	*ATM*	c.3190A>G p.Met1064Val	rs79431304/ VUS (BP4)	other
UB30	62 ER+/Her2+	*HMMR*	c.146-4G>A	rs199936654/ VUS (PM2)	uterus
UB33	49 ER+/Her2-	*RB1CC1*	c.4394C>T p.Thr1465Ile	Novel/ VUS (PM2, PP3)	other
		*ATM*	c.1683A>T p.Gln561His	Novel/ VUS (PM2)	
		*APC*	c.5105G>A p.Gly1702Glu	rs769273526/ VUS (PM2)	
UB35	57 TNBC	*PMS2*	c.746_753del p.Asp249Valfs*2	rs587782710/ P (PVS1, PS4, PM2)	-
		*MSH6*	c.1159G>A p.Asp387Asn	rs746532720/ VUS (PM2)	
		*HMMR*	c.1642C>A p.Gln548Lys	Novel/ VUS (PM2)	
UB36	59 ER-/Her2+	*CASR*	c.1190G>A p.Gly397Glu	rs1210105383/ LP (PM, PM5, PP, PP3)	ovary
UB43	39 ER+/Her2+	*CASP8*	c.268C>T p.Pro90Ser	rs1559350009/ VUS (PM2)	ovary
		*HMMR*	c.104C>T p.Pro35Leu	rs568662551/ VUS (PM2)	
UB44	50 ER-/Her2+	*SLC22A18*	c.28A>C p.Asn10His	rs575087578/ VUS (PM2)	-
UB48	56 ER+/Her2+	*ATM*	c.2838+9C>T	rs370160823/ VUS	-
UB49	46 TNBC	*BRCA2*	c.3861_3864del p.Asn1287Lysfs*5	rs886040500/ P (PVS1,PS4, PM2)	breast
UB52	37 ER+/Her2-	*ESR1*	c.433G>A p.Gly145Ser	rs201617046/ VUS (PM2)	-
UB53	35 ER-/Her2+	*ATM*	c.8805G>A p.Met2935Ile	rs772621438/ VUS (PP3)	-
		*MLH1*	c.776T>C p.Leu259Ser	rs56250509/ Pathogenic VUS (PM2, PP3)	
UB54	45 ER-/Her2+	*ACVR1B*	c.899G>C p.Gly300Ala	Novel/ VUS (PM2, PP2)	-
		*AR*	c.1009G>C p.Gly337Arg	rs1363782162/ VUS (PM2, PP3)	
UB55	56 ER-/Her2+	*XRCC3*	c.85C>T p.His29Tyr	rs546983534/ VUS (PM2)	-
UB59	49 ER+/Her2-	*BLM*	c.3558+3A>G	rs766386042/ VUS	-
		*MSH3*	c.3302+4A>C	rs779568504/ VUS	
UB60	41 ER+/Her2-	*MCPH1*	c.2257G>A p.Gly753Arg	rs587783737/ VUS (PM2)	breast
UB62	60 ER+/Her2-	*FGFR2*	c.1763A>G p.Tyr588Cys	rs770827652/ VUS (PM2, PP2)	other
		*PMS2*	c.229G>A p.Glu77Lys	rs751235177/ VUS (PM2)	
UB63	45 ER+/Her2-	*AR*	c.2182A>G p.Asn728Asp	Novel/ LP (PM1, PM2, PM5, PP2)	uterus
UB70	40 ER+/Her2+	*ARFGEF1*	c.2699-1G>A	rs200901179/ P (PVS1, PS4)	-
		*MLH1*	c.761A>G p.Lys254Arg	rs786202528/ VUS (PM2, PP3)	
UB76	59 ER-/Her2+	*RAD54L*	c.788G>A p.Gly263Glu	rs186059216/ VUS (PM2, PP3)	uterus
UB77	56 ER-/Her2+	*PRKN*	c.850G>C p.Gly284Arg	rs751037529/ P (PP1, PP3, PS3, PM2, PM3)	-
		*BRCA1*	c.3083G>A p.Arg1028His	rs80357459/ VUS (BP6)	
UB79	37 ER-/Her2+	*BRCA1*	c.1544_1550del p.Glu515Valfs*15	Novel/ P (PVS1, PS4, PM2)	-

ER: estrogen receptor; TNBC: triple-negative breast cancer (ER-/PR-/HER2-). LP: like pathogenic; P: Pathogenic; VUS: variant of uncertain significance. The symbol “*” is stop codon.

**Table 3:** Predictions from in silico software for missense variants.

Gen	cDNA	Protein	Varity	Mutation Assesor	Mutation Taster	SIFT	Polyphen2	DANN	Meta	Primate AI	Bayes Del	Geno Canyon	Fit Con	Prediction
*CHD8*	c.6472C>T	p.Arg2158Cys	D (0.73)	M (2.10)	D (1)	D (0.00)	D (0.84)	D (1)	D (0.91)	D (0.90)	D (0.5)	D (1)	D (0.7)	Pathogenic
*ATM*	c.5888A>G	p.Asp1963Gly	D (0.75)	M (2.52)	D (1)	D (0.01)	-	-	B (0.02)	U (0.57)	U (0.5)	D (1)	D (0.7)	Pathogenic
*LZTR1*	c.1646A>G	p.Asp549Gly	D (0.73)	M (2.16)	D (1)	D (0.01)	D (1)	-	U (0.50)	D (0.80)	D (0.2)	D (1)	D (0.7)	Pathogenic
*NF1*	c.4451C>G	p.Ser1484Cys	D (0.68)	M (0.02)	D (1)	D (0.01)	-	-	D (0.59)	U (0.78)	U (0.0)	D (0.97)	D (0.6)	Pathogenic
*WRN*	c.2977C>T	p.Arg993Cys	D (0.85)	H (3.80)	D (1)	D (0.00)	D (1)	D (1)	B (0.31)	B (0.41)	U (0.1)	D (1)	D (0.7)	Pathogenic
*MSH2*	c.2197G>A	p.Ala733Thr	D (0.87)	H (3.60)	D (1)	D (0.01)	D (0.96)	D (1)	D (0.81)	U (0.76)	D (0.2)	D (1)	D (0.7)	Pathogenic
*RAD54L*	c.788G>A	p.Gly263Glu	D (0.72)	M (2.32)	D (1)	D (0.01)	D (0.84)	D (1)	D (0.81)	U (0.63)	D (0.3)	D (1)	D (0.7)	Pathogenic
*CTNNB1*	c.1345C>T	p.Arg449Cys	D (0.72)	M (2.50)	D (1)	U (0.05)	D (0.64)	D (1)	D (0.56)	D (0.94)	D (0.2)	-	D (0.6)	Pathogenic
*BARD1*	c.1620A>T	p.Lys540Asn	D (0.42)	M (2.36)	D (0.97)	D (0.00)	D (0.97)	D (0.9)	D (0.64)	B (0.43)	B (−0.33)	B (0)	D (0.7)	Pathogenic
*MSH6*	c.944C>G	p.Ser315Cys	B (0.09)	L (1.61)	B (0.13)	U (0.04)	D (0.61)	D (0.9)	D (0.54)	B (0.27)	B (−0.21)	D (1)	D (0.6)	Conflict
*ATR*	c.4352G>A	p.Arg1451Gln	B (0.07)	N (−0.95)	D (1)	B (1)	B (0.01)	D (1)	B (0.01)	B (0.36)	B (−0.68)	D (1)	D (0.7)	B
*KLLN*	c.250G>C	p.Gly84Arg	B (0.11)	N (0)	B (0)	B (0.59)	U (0.27)	D (0.8)	B (0.04)	B (0.31)	B (−0.53)	-	B (0.0)	B
*MLH1*	c.776T>C	p.Leu259Ser	D (0.92)	L (1.84)	D (1)	D (0)	D (1)	D (1)	B (0.29)	U (0.67)	D (0.2)	D (1)	D (0.7)	Pathogenic
*TP53*	c.1024C>G	p.Arg342Gly	D (0.81)	M (2.40)	B (0)	U (0.05)	-	D (1)	D (0.77)	B (0.27)	U (−0.08)	B (0)	D (0.7)	Conflict
*RAD54L*	c.866G>A	p.Ser289Asn	B (0.06)	N (0.56)	D (0.74)	B (0.21)	B (0.01)	D (0.9)	D (0.55)	B (0.44)	B (−0.37)	B (0)	D (0.7)	Conflict
*SLC22A18*	c.604A>G	p.Ile202Val	B (0.06)	M (2.16)	B (0.43)	B (0.12)	U (0.25)	D (0.9)	B (0.33)	B (0.45)	B (−0.41)	B (0.34)	D (0.7)	B
*APC*	c.5290C>G	p.Gln1764Glu	B (0.1)	L (0.81)	B (0)	D (0.01)	B (0.29)	D (0.9)	B (0.49)	B (0.37)	B (−0.21)	D (1)	D (0.7)	B
*ATM*	c.3190A>G	p.Met1064Val	B (0.21)	M (2.14)	B (0)	U (0.07)	B (0)	B (0.4)	B (0.16)	B (0.27)	B (−0.40)	B (0)	D (0.6)	B
*RB1CC1*	c.4394C>T	p.Thr1465Ile	D (0.47)	N (0)	D (1)	D (0.00)	-	D (1)	B (0.06)	D (0.79)	B (−0.34)	D (1)	D (0.7)	Pathogenic
*ATM*	c.1683A>T	p.Gln561His	B (0.04)	L (1.60)	B (0)	B (0.33)	-	D (0.9)	B (0.13)	B (0.33)	B (−0.45)	D (0.60)	D (0.7)	B
*APC*	c.5105G>A	p.Gly1702Glu	B (0.09)	N (0)	B (0)	B (0.44)	B (0)	D (0.9)	B (0.39)	B (0.33)	B (−0.32)	B (0)	D (0.7)	B
*MSH6*	c.1159G>A	p.Asp387Asn	B (0.21)	N (0.63)	D (1)	B (0.27)	-	D (0.9)	B (0.40)	B (0.32)	B (−0.38)	D (1)	D (0.6)	Conflict
*HMMR*	c.1642C>A	p.Gln548Lys	B (0.06)	L (1.52)	B (0.08)	B (0.12)	-	D (0.8)	B (0.02)	B (0.37)	B (−0.62)	D (0.97)	D (0.7)	B
*CASP8*	c.268C>T	p.Pro90Ser	B (0.28)	M (2.02)	B (0.46)	D (0.01)	-	D (1)	D (0.69)	B (0.41)	U (−0.14)	D (1)	D (0.7)	Conflict
*HMMR*	c.104C>T	p.Pro35Leu	B (0.16)	M (2.56)	D (1)	U (0.09)	D (1)	D (1)	B (0.07)	U (0.52)	B (−0.36)	D (1)	D (0.7)	Conflict
*ESR1*	c.433G>A	p.Gly145Ser	B (0.12)	L (0.26)	D (0.96)	B (0.24)	D (0.87)	D (0.9)	B (0.02)	U (0.55)	B (−0.47)	D (1)	B (0.4)	B
*ATM*	c.8805G>A	p.Met2935Ile	D (0.54)	M (2.69)	D (1)	U (0.05)	D (0.90)	D (0.9)	D (0.66)	U (0.69)	B (−0.07)	D (1)	D (0.7)	Pathogenic
*MLH1*	c.776T>C	p.Leu259Ser	D (0.92)	L (1.84)	D (1)	D (0)	D (0.97)	D (0.9)	B (0.29)	U (0.67)	D (0.2)	D (1)	D (0.7)	Pathogenic
*ACVR1B*	c.899G>C	p.Gly300Ala	-	-	-	D (0)	-	B (0.5)	D (0.52)	B (0.44)	B (−0.37)	D (1)	D (0.5)	Conflict
*AR*	c.1009G>C	p.Gly337Arg	B (0.15)	-	B (0.2)	B (0.17)	-	D (1)	D (0.92)	U (0.60)	D (0.46)	D (1)	-	Conflict
*XRCC3*	c.85C>T	p.His29Tyr	B (0.04)	M (1.94)	B (0)	B (0.11)	U (0.21)	D (0.8)	B (0.08)	B (0.22)	B (−0.41)	D (1)	-	B
*MCPH1*	c.2257G>A	p.Gly753Arg	B (0.25)	M (2.35)	D (0.98)	U (0.06)	D (1)	D (1)	B (0.05)	B (0.45)	B (−0.29)	D (1)	D (0.7)	Conflict
*FGFR2*	c.1763A>G	p.Tyr588Cys	D (0.68)	L (1.78)	D (1)	D (0.00)	-	D (0.9)	D (0.58)	D (0.82)	U (0.02)	D (1)	D (0.7)	Pathogenic
*PMS2*	c.229G>A	p.Glu77Lys	D (0.72)	L (1.14)	D (1)	D (0.00)	D (0.98)	D (0.9)	B (0.33)	U (0.58)	U (0.00)	D (1)	D (0.7)	Pathogenic
*MLH1*	c.761A>G	p.Lys254Arg	D (0.58)	L (1.77)	D (1)	U (0.03)	-	D (1)	B (0.33)	U (0.60)	U (0.13)	D (1)	D (0.7)	Pathogenic
*RAD54L*	c.788G>A	p.Gly263Glu	D (0.72)	M (2.32)	D (1)	D (0.00)	D (0.84)	D (1)	D (0.81)	U (0.63)	D (0.39)	D (1)	D (0.7)	Pathogenic
*BRCA1*	c.3083G>A	p.Arg1028His	B (0.01)	N (−1.15)	D (0.65)	B (0.29)	B (0)	D (0.8)	B (0.06)	B (0.18)	B (−0.31)	D (1)	D (0.7)	B

B: benign; D: deleterious; H: high; L: low; M: medium; N: neutral; U: uncertain.

**Table 4:** Prediction of splice variants based on the Human Splicing Finder (HSF) and MaxEnt Scan.

ID	Gene	Variant	HSF (%)	MaxEnt (%)	Prediction
UB21	*SPTBN1*	c.109+1G>T	−28.00	−83.27	Broken donor site
	*NF1*	c.888+5G>A	-	-	-
UB23	*NF1*	c.888+5G>A	-	-	-
UB30	*HMMR*	c.146-4G>A	-	-	-
UB48	*ATM*	c.2838+9C>T	-	-	-
UB59	*BLM*	c.3558+3A>G	−22.92	−74.69	Broken donor site
	*MSH3*	c.3302+4A>C	−12.16	−44.85	Broken donor site

## Data Availability

Data sharing is not applicable to this article.
